# If a fish can pass the mark test, what are the implications for consciousness and self-awareness testing in animals?

**DOI:** 10.1371/journal.pbio.3000021

**Published:** 2019-02-07

**Authors:** Masanori Kohda, Takashi Hotta, Tomohiro Takeyama, Satoshi Awata, Hirokazu Tanaka, Jun-ya Asai, Alex L. Jordan

**Affiliations:** 1 Laboratory of Animal Sociology, Department of Biology and Geosciences, Osaka City University, Osaka, Japan; 2 Department of Biosphere-Geosphere Science, Okayama University of Science, Okayama, Japan; 3 Institute of Ecology and Evolution, Department of Behavioural Ecology, University of Bern, Hinterkappelen, Switzerland; 4 Department of Collective Behaviour, Max Planck Institute for Ornithology, Konstanz, Germany; Emory University, UNITED STATES

## Abstract

The ability to perceive and recognise a reflected mirror image as self (mirror self-recognition, MSR) is considered a hallmark of cognition across species. Although MSR has been reported in mammals and birds, it is not known to occur in any other major taxon. Potentially limiting our ability to test for MSR in other taxa is that the established assay, the mark test, requires that animals display contingency testing and self-directed behaviour. These behaviours may be difficult for humans to interpret in taxonomically divergent animals, especially those that lack the dexterity (or limbs) required to touch a mark. Here, we show that a fish, the cleaner wrasse *Labroides dimidiatus*, shows behaviour that may reasonably be interpreted as passing through all phases of the mark test: (i) social reactions towards the reflection, (ii) repeated idiosyncratic behaviours towards the mirror, and (iii) frequent observation of their reflection. When subsequently provided with a coloured tag in a modified mark test, fish attempt to remove the mark by scraping their body in the presence of a mirror but show no response towards transparent marks or to coloured marks in the absence of a mirror. This remarkable finding presents a challenge to our interpretation of the mark test—do we accept that these behavioural responses, which are taken as evidence of self-recognition in other species during the mark test, lead to the conclusion that fish are self-aware? Or do we rather decide that these behavioural patterns have a basis in a cognitive process other than self-recognition and that fish do not pass the mark test? If the former, what does this mean for our understanding of animal intelligence? If the latter, what does this mean for our application and interpretation of the mark test as a metric for animal cognitive abilities?

**Editor’s note:**

This Short Report received both positive and negative reviews by experts. The Academic Editor has written an accompanying Primer that we are publishing alongside this article (https://doi.org/10.1371/journal.pbio.3000112). The linked Primer presents a complementary expert perspective; it discusses how the current study should be interpreted in the context of evidence for and against self-awareness in a wide range of animals.

## Abstract 2

A marine fish, the cleaner wrasse, appears to perform all the appropriate behavioural responses during the mirror, or mark test. Does this mean that cleaner wrasse are self-conscious? Or that our interpretation of what the mark test means needs to be updated?

## Author summary

The ability to perceive and recognise a reflected mirror image as self is considered a hallmark of cognition across species. Here, we show that a fish, the cleaner wrasse, shows behavioural responses that can be interpreted as passing the mark (or mirror) test, a classic test for self-awareness in animals. We ask whether these behaviours should be taken as evidence that fish are self-aware or whether the test itself needs to be revised. In particular, we interrogate whether tests such as these can be reliably employed in animals as divergent from humans as fish and how we might understand cognition in nonprimates.

## Introduction

The mark test, in which a coloured mark is placed on a test subject in a location that can only be viewed in a mirror reflection, is held as the benchmark behavioural assay for assessing whether an individual has the capacity for self-recognition [1,2]. In human infants, approximately 65% of individuals pass the mark test by 18 mo of age by touching the mark with their hands while viewing their reflection [3], although some individuals pass earlier, and some never pass. Accumulating reports claim that many other animal species also pass the mark test, including chimpanzees [1], elephants [4], dolphins [5,6], and corvids [7], while many other species are apparently unable to pass the test [8] (but see [9–11]). Nevertheless, the interpretation of these results is subject to wide debate, and the certainty with which behavioural responses during the mirror test can be taken as evidence of self-awareness in animals is questioned [8,12,13]. This problem is exacerbated when the taxonomic distance increases between the test species and the primate taxa for which the test was initially designed. For instance, can the behavioural results recorded for chimpanzees during the mirror test be meaningfully compared with the responses of a bird? If so, does this mean a bird that passes the mirror test is self-aware? More generally, if we are interested in understanding and comparing cognition and problem solving across taxa, can we assume that equivalent behaviours represent equivalent underlying cognitive processes? With particular reference to the mark test, here we explore what forms of behaviour in fish could be taken as evidence of self-awareness and whether the same conclusions that have been drawn in other taxa can also be drawn for fish.

Given that the mark test as designed for primates relies on hand gestures towards the marked region and changes in facial expression, we also ask whether it is even possible to interpret the behaviour of divergent taxonomic groups during the mark test in the same way as for the taxa for which the test was initially designed. If not, the usefulness of the mark test across taxa must be questioned, as should our confidence in sharp divisions in cognitive abilities among taxa. To explore these questions, we here test whether a fish, the cleaner wrasse *L*. *dimidiatus*, displays behavioural responses that can be interpreted as passing the mark test. We then ask what this may mean for our understanding of self-awareness in animals and our interpretation of the test itself.

To date, no vertebrate outside of mammals and one bird species has passed the mark test. This is despite many species in other vertebrate classes, such as fish, showing sophisticated cognitive capacities in other tasks [14–17], including transitive inference [18,19], episodic-like memory [20], playing [21], tool use [22,23], prediction of the behaviour of others by using one’s own experience during coordinated hunting [24,25], cooperating to warn about predators [26,27], and cooperative foraging [28]. These studies reveal that the perceptual and cognitive abilities of fish often match or exceed those of other vertebrates [15,17] and suggest the possibility that the cognitive skills of fish could more closely approach those found in humans and apes [14,16,17,24,28]. Clearly, a claim such as this requires rigorous testing to be held up and an accepted framework in which the results of any test can be interpreted.

It can be challenging to employ standardised cognitive tests across species when performance in the test depends on specific behavioural responses that are not present in all taxa or, perhaps more importantly, that are difficult for human observers to objectively interpret. This may be the case for the mark test, which has been specifically designed to suit the behavioural repertoire of primates [1,2]. Animals that cannot directly touch the marks used in mirror self-recognition (MSR) tests may therefore be inherently poor test candidates [2,5,29] regardless of their cognitive abilities, making direct comparison across taxa challenging [30*–*33]. Manta rays (Chondrichthyes), for instance, show unusual behaviour on exposure to a mirror, and it has been suggested these are self-directed behaviours in response to seeing their own reflection [34], although no mark test was performed and this interpretation is contested [35,36]. This controversy highlights the need to ask what type of behavioural response would be taken as evidence of contingency testing, self-directed behaviour, or self-exploration in an animal with such divergent morphology and behaviour from typical test species.

To make a comparison across taxa, initially it may be useful to choose species with perceptual abilities and a behavioural repertoire that allow them to respond to coloured marks placed on the body (this is not a given when the sensory systems of animals differ so greatly) and do so in a manner that can be effectively interpreted by a human observer. The cleaner wrasse, *L*. *dimidiatus*, is potentially such a species because it forms mutualistic relationships with larger client fish by feeding on visually detected ectoparasites living on the skin of the clients [37]. Therefore, the cleaner wrasse has sensory and cognitive systems that are well equipped for visually detecting spots of unusual colour on the skin surface, as well as the motivation to behaviourally respond to marks. Importantly, the natural response to removing parasites from clients—directly biting them—would result in cleaner wrasse biting at the mirror surface rather than performing self-directed behaviour, which would constitute failing the test. The role of hard-wired behavioural responses to parasites could therefore be ruled out. Additionally, this species is highly social, interacting with the same individuals repeatedly over long periods of time, and has sophisticated cognitive abilities, including tactical deception [38–40], reconciliation [41], and the ability to predict the actions of other individuals [41,42]. These are traits requiring cognitive abilities that may be correlated with the ability for self-recognition [e.g., 16,29,43–45].

During the mark test, animals must visually locate a mark in a mirror image that cannot be viewed directly. Given their sensory biology, it is reasonable to predict the wrasse will notice the coloured marks and that marks may generate an attentional response that culminates in a removal attempt [46–47]. However, lacking hands or trunks, any attempt to remove or interact with the mark would necessarily take a different form than is seen in many other taxa. Fortunately for the question at hand, many fish display a characteristic self-directed behaviour that functions to remove irritants and/or ectoparasites from the skin surface, termed glancing or scraping [48,49]. Similarly, mammals such as dolphins that lack hands may scrape their own bodies, and this behaviour has been interpreted as self-directed behaviour during application of the mark test in those species [29,50]. We therefore consider the cleaner wrasse to possess the prerequisite sensory biology and behavioural repertoire to adequately implement the mark test and here use a modified experimental design to test for MSR in a fish. Importantly, this experiment allows us to ask a broader question of whether the criteria that are accepted as evidence for MSR in mammals and birds can be applied to other taxa, and if these fish fulfil these criteria, what it means for our interpretation of the test itself.

**Fig 1:**
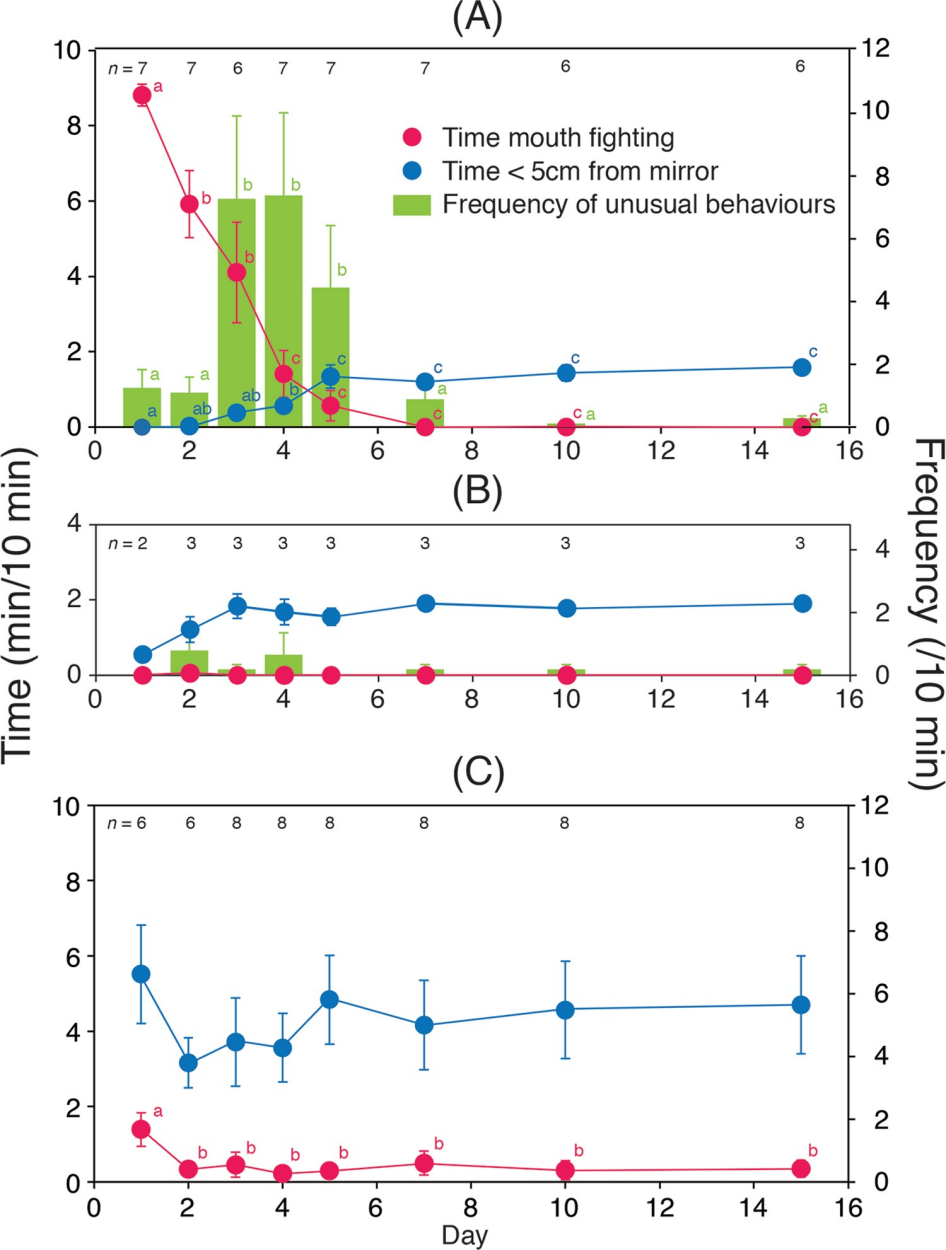
Responses of cleaner wrasse to the mirror and to real fish across a clear divider. (A) Change in social responses towards the mirror. Mean ± SE for time spent mouth fighting (red), time spent within 5 cm of the mirror without being aggressive (blue), and frequency of mirror-testing behaviours/10 min (green). Superscript labels a, b, and c denote statistical differences. Statistical results for daily changes in time spent mouth fighting, LMM, c_7_^2^ = 91.87, *P* < 0.0001; time spent in front of the mirror, LMM, c_7_^2^ = 64.63, *P* < 0.0001; and changes in the number of mirror-testing behaviours, GLMM, c_7_^2^ = 137.08, *P* < 0.0001. (B) Change in social responses towards the mirror for fish that failed to pass through prephases of the test. (C) Change in social responses to conspecific fish over 2 wk: statistical results for daily changes in time spent mouth fighting, LMM, df = 7, *χ*^2^ = 27.36, *P* = 0.0003, and time spent in front of the mirror, LMM, df = 7, *χ*^2^ = 9.09, *P* = 0.25; no idiosyncratic behaviours were observed in this condition. Underlying data for this figure can be found in S1 Data. df, degrees of freedom; GLMM, generalised linear mixed model; LMM, linear mixed model.

Prior to the provisioning of a coloured mark, transitions among three behavioural phases after initial exposure to a mirror are typically observed [1,4,5,6]. These transitions among behavioural phases are interpreted as additional evidence of self-recognition, although in themselves do not constitute passing the mark test, which specifically requires mark removal attempts [1,4]. The first phase (i) is a social reaction towards the mirror, apparently as a consequence of the reflection being perceived as an unknown conspecific. In phase (ii), animals begin to repetitively perform idiosyncratic behaviours that are rarely observed in the absence of the mirror. These behaviours are interpreted as contingency testing between the actions of the subject and the behaviour of the reflection [e.g., 1,4]. In phase (iii), the subject begins to examine their reflection and uses the mirror to explore their own body in the absence of aggression and mirror-testing behaviour [1,4,5]. Finally, a coloured mark is applied, and observations of removal attempts are recorded. Here, we first tested whether the cleaner wrasse pass through all three behavioural phases upon exposure to a mirror (Fig 1) and then provided a mark using subcutaneous injections of transparent or pigmented elastomer to test for removal attempts.

## Results and discussion

Prior to starting the experiments, the focal fish swam around the tank and showed no unusual reactions to the covered mirror. Immediately after initial exposure to the mirror, seven of 10 fish responded aggressively to their reflection, attacking it and exhibiting mouth fighting (Fig 1 and S1 Video [45,46]), suggesting that the focal fish viewed the reflection as a conspecific rival. The frequency of mouth fighting was highest on day 1 and decreased rapidly thereafter, with zero occurrences by day 7 and almost no aggression throughout the remainder of the experimental period (Fig 1A; cf. a similar decrease in aggression seen in chimpanzees and shown in Figure 2 of [1]). This initially high and subsequently decreasing aggression is consistent with phase (i) of the mark test as reported in other taxa.

As mouth fighting towards the mirror reflection decreased, the incidence of atypical behaviours (e.g., swimming upside-down, a highly unusual behaviour typically never observed in cleaner wrasse; Table 1 and S2 and S3 Videos) significantly increased and was highest on days 3 to 5 (Fig 1A). On days 3 and 4, the estimated average frequency of these atypical behaviours across the seven individuals was extremely high—36 times per hour. Each of these atypical behaviours was of short duration (≤1 s), often consisting of rapid actions with sudden onset within 5 cm of the mirror, and could be loosely grouped into five types (Table 1). While it is possible to interpret these behaviours as a different form of aggression or social communication, they have not been recorded in any previous studies of social behaviour in this species [46] and were not likely to be part of a courtship display because all of the subject fish were females. Moreover, we did not observe these behaviours in our own control experiments when presenting a conspecific across a clear divide (Fig 1C), further demonstrating they were unlikely to be forms of social communication.

**Table 1:** Total occurrence of unusual behaviours shown by seven fish during 20-min observation periods during the first 5 d after presentation of the mirror.

Individual fish	Occurrence of behaviour a	Occurrence of behaviour b	Occurrence of behaviour c	Occurrence of behaviour d	Occurrence of behaviour e	Total occurrence of behaviours
#1	2	0	4	**39**	0	45
#2	0	0	**30**	0	0	30
#3	**54**	0	0	0	0	54
#7	15	0	0	0	**35**	50
#13	**33**	0	0	0	0	33
#20	**31**	17	2	0	2	52
#21	2	0	2	0	**6**	10
Total	137	17	38	39	43	274

Number of atypical behaviours shown by seven fish (individuals #1, #2, #3, #7, #13, #20, and #21) for each 20-min observation period in the first 5 d after presenting the mirror: (a) dashing along the mirror, (b) dashing with head in contact with the mirror, (c) dashing and stopping, (d) upside-down approach, and (e) quick ‘dance’. The most frequent mirror-testing behaviour of each fish is in bold. Descriptions of behaviour are as follows. Dashing along the mirror: rapid dashing along the mirror surface in a single direction for 10–30 cm. Fish do not swim directly against or make contact with their mirror reflection. Dashing along the mirror with the head in contact with the mirror: the head of the fish was always in contact with the mirror during dashing. Dashing and stopping: fish rapidly dashed towards the mirror reflection but stopped before contact with the mirror. Upside-down approach: fish swim in an upside-down posture while approaching the mirror. Quick ‘dance’: fish spread all of their fins and quickly arched and quivered the body several times over ca. 1 s at a distance 5–10 cm from the mirror; no dashing to the mirror is observed.

These atypical behaviours were individually specific, with each fish performing one or two types of behaviour (Table 1; Fisher’s exact probability test for count data with simulated *P* value based on 2,000 replicates of *P* = 0.0005). Crucially, these behaviours occurred only upon exposure to the mirror and were not observed in the absence of the mirror (i.e., before mirror presentation) or during conspecific controls. Almost all of the behaviours ceased by day 10 (Fig 1A) and were rarely observed thereafter. These behaviours were different from the previously documented contingency-testing behaviours of great apes, elephants, and magpies [1,4,7], but given the taxonomic distance between them, this could hardly be otherwise. While primates and elephants may perform more anthropomorphic behaviours such as changing facial expression or moving the hands, legs, or trunk in front of the mirror, wrasse and other fishes cannot perform behaviours that are so easily interpreted by a human observer. Nevertheless, behaviours such as upside-down swimming are indeed unusual for a healthy fish and could represent alternative indices of contingency that are within the behavioural repertoire of the study species. Moreover, the atypical movements observed in cleaner wrasse were consistent with behaviour previously interpreted as contingency testing in other species [1,4,5,7] in that these behaviours were atypical and idiosyncratic, repetitive, displayed only in front of the mirror, absent in the absence of a mirror, shown after a phase of initial social (here aggressive) behaviour, displayed over a short period of time, and distinct from aggressive behaviour. Although we reserve judgement as to whether these behaviours should unequivocally be interpreted as evidence that these fish are examining and perceiving the reflection as a representation of self, we nevertheless argue that on an objective basis, these behaviours fulfil the criteria as presented for contingency testing and are consistent with phase (ii) of MSR as presented for other taxa [1,4,5,7].

In phase (iii), species that pass the mark test increase the amount of time spent in front of the mirror in nonaggressive postures, apparently visually exploring their own bodies [1,4,5,7]. This interpretation is again rife with pitfalls because it requires an assessment of the intentionality of nonhuman animal behaviour. An agnostic approach is to simply measure the amount of time animals spend in postures that could reflect the body in the mirror [2], giving an upper measurement of the time in which animals could observe their reflection while making no inferences about the intentionality of the act. We observed an increase in the amount of time spent in nonaggressive postures while close to the mirror (distance of <5 cm), peaking on day 5 after mirror presentation and remaining consistently higher than days 1 to 4 (Wilcoxon sign-ranked test, *T* = 36, *P* = 0.008; Fig 1A). Although we did not observe directed viewing behaviour as seen in chimpanzees and elephants, this would in any case be difficult given challenges of assessing gaze direction in animals like fish (although see [45] for a recent technological solution). We therefore consider that in terms of time spent in postures that would facilitate viewing the mirror reflection, this behaviour was consistent with phase (iii) of MSR.

Species with MSR distinguish their own reflection from real animals viewed behind glass [e.g., 29]. When we exposed naïve cleaner wrasse to conspecifics behind glass, we observed fundamentally different responses towards their mirror image (S1 Text). Aggressive behaviour frequency towards real fish was generally low yet did not diminish appreciably during the 2-wk testing period (Fig 1C). Time spent within 5 cm of the glass in the presence of conspecifics was also higher than that in the presence of the mirror. Importantly, no atypical or idiosyncratic behaviour (that might be considered contingency testing) was exhibited towards conspecifics. These behaviours were only observed upon exposure to the mirror. Similar to many previous MSR studies [1,4,5,7], not all individuals we tested passed through every phase of the test. After the initial presentation of the mirror, three fish showed low levels of aggression and rarely performed atypical behaviours during period E1 (Fig 1B). Instead, these three individuals spent relatively longer periods in front of the mirror, as is typically observed during phase (iii), and we conclude these fish failed the test (but see S1 Text for an alternative explanation).

In the second part of the experiment, we used a modified standard mark test protocol to assess reactions to visible (pigmented) or sham (transparent) marks. We used subcutaneously injected elastomer (see Materials and methods) to apply a small amount of colour below the skin surface, a widely used procedure that has been repeatedly shown not to affect fish behaviour [51–54, Northwest Marine Technology]. Moreover, the combined use of coloured and transparent sham marks provided an internal control for the effects of application, including irritation or tactile sensations around the marking site. Nevertheless, the procedure certainly resulted in higher tactile stimulation than, e.g., paint marks on elephant skin, necessarily so because of the requirements of provisioning marks in the aquatic environment and on animals covered in a protective mucus coating. We must therefore consider recent studies showing that visual–somatosensory training induced self-directed behaviour in rhesus monkeys [10,11] that could not be achieved through visual stimuli alone. Our study differs in that we do not provide direct somatic stimulation during the mark test and that we observed no response during our sham-mark phases, which also used a subcutaneous injection. However, given the nature of the mark application, we cannot rule out that a combination of visual and tactile cues produces the behavioural responses we describe, and our test might therefore be considered more similar to the modified tactile–visual mark test than the original mark test.

Fish were marked at night while under anaesthesia, and they swam normally the next morning in the no-mirror condition. After the initial settlement period ‘E1’ (i.e., the initial 2 wk of phases i–iii), we evaluated behaviour during periods ‘E2’ (no mark), ‘E3’ (injection with transparent sham mark), ‘E4’ (injection with coloured mark with no mirror present), and ‘E5’ (coloured mark with mirror present) during a subsequent 2-wk period. The sham and coloured marks were applied on the right side of the head of two fish, on the left side of the head of two other fish, and under the throat in a further four fish; these areas were only visible in the mirror. Each mark was in the form of a small brown mark with the intention of mimicking a natural ectoparasite in colour, size, and shape.

We first examined whether fish assumed postures in front of the mirror that would reflect the marked site by categorising all body postures performed within 5 cm of the mirror into three categories: postures exposing the right side of the head to the mirror, postures exposing the left side of the head, and frontal–vertical postures exposing the head, throat, and underside to the mirror. These postures would reflect the right face mark, the left face mark, and the throat mark, respectively. We predicted that if fish were attempting to observe the coloured marks on body parts reflected in the mirror, they would assume postures that facilitated this observation of the mark significantly more frequently during E5 (mirror, colour mark) than in E2 (mirror, no mark) or E3 (mirror, transparent sham mark). Two independent analyses of the videos were conducted (by MK and JA), as well as two further blind analyses by unrelated researchers of a subset (15%) of the videos; the frequencies were highly correlated between the analyses (*r* = 0.988).

**Fig 2:**
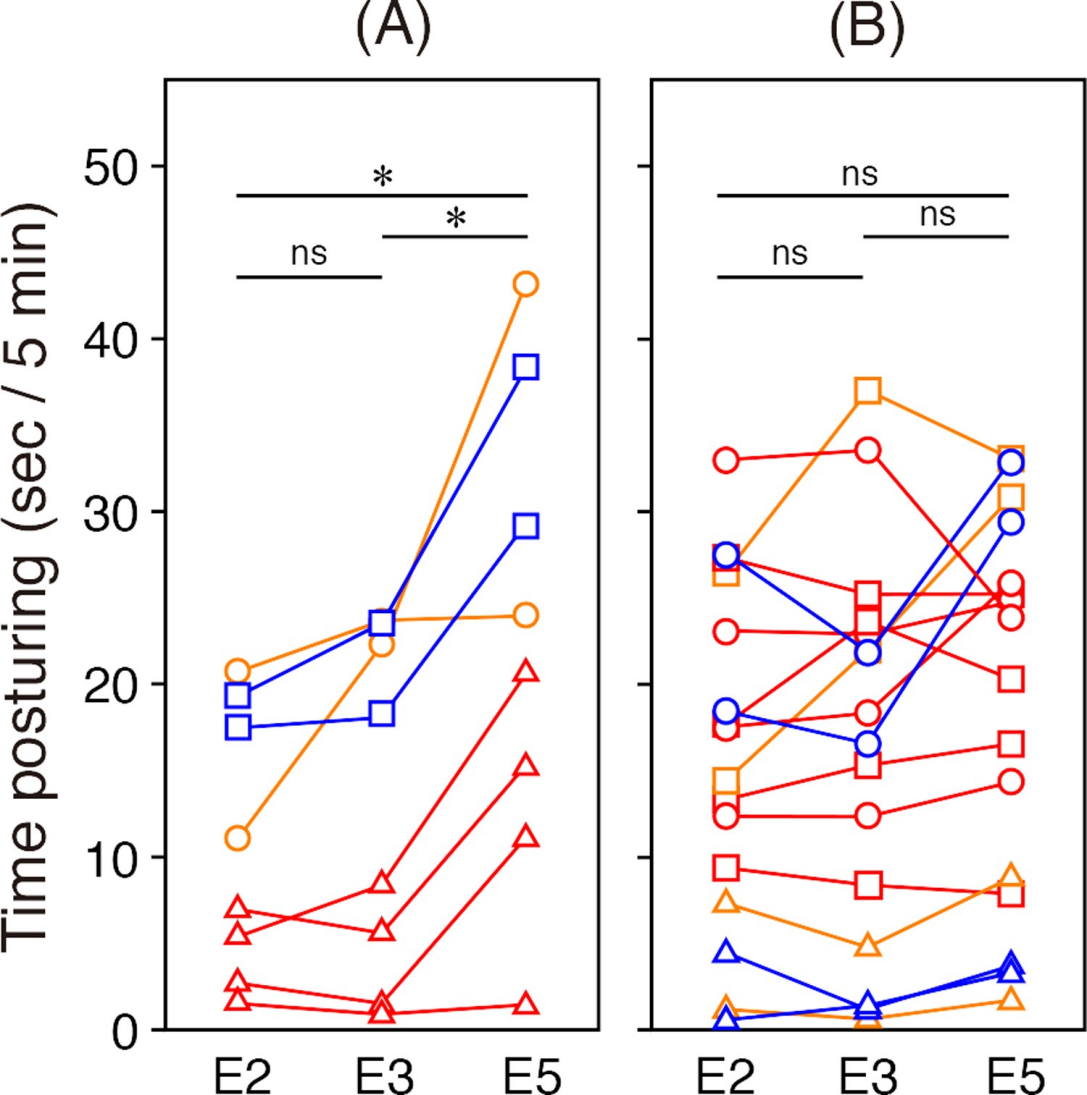
Mark locations and time spent in postures facilitating viewing of the marked site. (A) Time spent in postures that reflect the marked location (i.e., the ‘correct’ side). Repeated-measures ANOVA, main effect of sequences: *F* = 12.09, *P* = 0.016; marked position: *F* = 19.06, *P* = 0.005; sequence × marked position: *F* = 0.70, *P* = 0.54. *P < 0.05 (*n* = 8). (B) Time spent in postures that reflect the unmarked locations. Repeated-measures ANOVA, sequence: *F* = 2.54, *P* = 0.12; marked position: *F* = 13.15, *P* = 0.0008; sequence × marked position: *F* = 0.99, *P* = 0.42. Underlying data for this figure can be found in S1 Data. ANOVA, analysis of variance; ns, not significant. Line colours: marked site on right side of head (orange), left side of head (blue), or throat (red). Node shapes: postures reflecting right side of head (circle), left side of head (square) or throat (triangle).

Posturing behaviours that would reflect the marked sites during periods E2 and E3 were infrequent, and all sides were presented equally (Fig 2A) in all fish except fish #7 (Table 2), suggesting the marking procedure itself had minimal effect on posturing behaviour. In contrast, time spent posturing while viewing the marked sites was significantly higher in the colour-marked (E5) versus no- (E2) and sham-marked (E3) periods (Fig 2A). This pattern held for all individuals except fish #2, regardless of the sites marked (Table 2). Note that no comparisons to E4 can be made with respect to observations of reflections because no mirror was present during that period. Moreover, the time spent in postures reflecting the two remaining unmarked sites (e.g., right side of head and throat for a fish marked on the left side of the head) were not different among periods (Fig 2B). Taken together, these findings demonstrate that cleaner wrasse spend significantly longer in postures that would allow them to observe colour-marked sites in the mirror reflection, and in previous studies on dolphins, similar patterns of activity were considered to constitute self-directed behaviour [5]. These reactions also demonstrate that tactile stimuli alone are insufficient to elicit these responses because they were only observed in the colour mark/mirror condition. Rather, direct visual cues or a combination of visual and tactile stimuli are essential for posturing responses in the mark test.

**Table 2:** Comparison of time spent in postures in which the marked site was reflected between experimental periods of the mark test.

Marked Sites	E2 versus E3	E2 versus E5	E3 versus E5
Throat mark			
#1	0.04* (5.5)	**0.004** (0.0)	**0.004** (0.0)
#4	0.27 (9.0)	**0.004** (0.0)	**0.004** (0.0)
#20	0.47 (13.5)	**0.020** (4.0)	**0.020** (2.0)
#21	0.08 (7.0)	**0.043** (5.5)	0.25 (11.0)
Right head mark			
#2	0.31 (9.5)	0.15 (14.5)	0.93 (14.5)
#7	0.01* (0.0)	**0.004** (0.0)	**0.010** (0.0)
Left head mark			
#5	0.71 (13.0)	**0.004** (1.0)	**0.011** (4.0)
#6	0.31 (9.0)	**0.011** (4.0)	**0.024** (4.0)

Statistical tests (Mann–Whitney *U* test) at the level of the individual between E2 (no mark) and E3 (sham mark), between E2 and E5 (coloured mark), and between E3 and E5. *P* values, with the *U* statistic in parentheses, are reported. *P* values < 0.05 are in bold.

*Time in E2 > time in E3 in fish #1, but E2 < E3 in fish #7.

Although they cannot touch their own bodies directly, many species of fish scrape their bodies on a substrate to remove irritants and/or ectoparasites from the skin surface [48,49]. When we marked fish with brown-pigmented elastomer on the lateral body surfaces in locations that could be viewed directly (i.e., without the mirror), we observed increased scraping behaviour on the site of the mark (S1 Fig). We therefore hypothesised that when marked in locations that could only be seen with the aid of a mirror, wrasse would similarly scrape their bodies in an attempt to remove the marks. We hypothesised this would occur in fish after viewing these marks in the mirror, and crucially, that they would not scrape transparent sham marks nor coloured marks in the absence of a mirror. Like many natural behaviours, some scraping of the body flanks was observed outside the mirror condition in our studies and was also difficult to distinguish from face scraping. Because of this, we restricted our analysis only to throat scraping and took this behaviour as the only evidence of a putative self-directed behaviour because it was never observed outside the period E5 in any of the subject fish.

**Fig 3:**
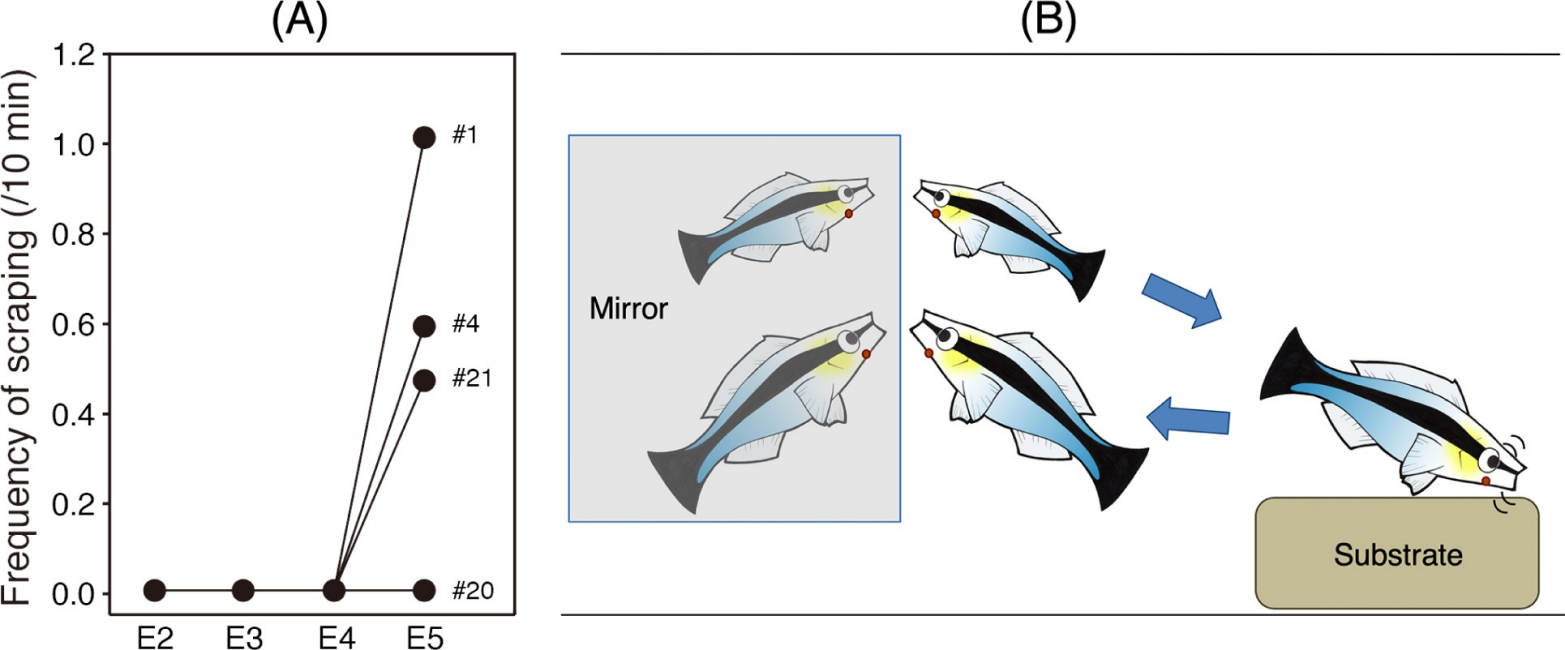
Frequency of throat-scraping behaviour throughout experimental phases. (A) Frequency of throat-scraping behaviour of the four throat-marked fish during periods E2, E3, E4, and E5. (B) Schematic sequence of posturing, throat-scraping behaviour, and then posturing again in positions that reflect the throat. Underlying data for this figure can be found in S1 Data.

After throat marking, three out of four fish scraped their throats against the substrate upon exposure to the mirror during period E5 (Fig 3 and S4 Video), but none of the four fish exhibited this behaviour during E2–E4 (control, transparent mark, and coloured mark without a mirror). This is a ratio comparable to other species tested previously; one of three Asian elephants passed the test [4], as did two of five magpies [6]. In total, we observed 37 separate instances of throat scraping during E5 (15 for fish #1, 16 for fish #4, 6 for fish #21; Friedman test, *χ*^2^ = 9.0, degrees of freedom (df) = 3, *P* = 0.029; binomial test within individuals, E2, E3, and E4 versus E5: 0 versus 15 scrapings, *P* < 0.0001 in fish #1, 0 versus 16 scrapings, *P* < 0.0001 in fish #4, 0 versus 6 scrapings, *P* = 0.031 in fish #21). The motivation for scraping the mark is potentially to remove a perceived ectoparasite, which these wild-caught fish would have experienced previously. Crucially, these scraping attempts are the opposite to what would be expected if cleaner wrasse were ‘hard-wired’ to remove anything resembling a parasite. In this case, we would expect fish to attempt to bite at the mark itself as though they were cleaning a client. To control for this possibility, we placed identical marks on the surface of the mirror itself but observed no attempts to remove these marks nor any scraping behaviour (S1 Text), demonstrating the scraping behaviour during the mark test was not a consequence of an innate response to marks that resemble parasites. Given that scraping behaviour is accepted as being self-directed in mammals during the mark test [29,50], we similarly interpret this behaviour as being self-directed in fish. Alternative interpretations risk introducing subjective taxonomic biases, setting moving goal posts, and precluding scientific comparisons among certain taxa. If scraping behaviour is therefore interpreted as self-directed, these results constitute compelling evidence that three of the four throat-marked fish passed through all prephases of the test and subsequently attempted to remove visually perceived coloured marks from their bodies after viewing them in the mirror. By extension and comparison to similar mark test studies, this leads to the crucial question of whether fish are aware that the mirror reflection is a representation of their own body.

The mark test is a controversial assessment of animal cognition [8] and perhaps even more so when applied to fish, a taxonomic group considered by some to have lesser cognitive abilities than other vertebrate taxa. Nevertheless, we provide compelling evidence that cleaner wrasse show behavioural responses that can be reasonably interpreted as passing through all stages of the mark test and attempt to remove a mark only when it is able to be viewed in the mirror (Fig 3). The results we present here will by their nature lead to controversy and dispute, and we welcome this discussion. We consider three possible interpretations of our results and their significance for understanding the mark test. The first (I) is that the behaviours we document are not self-directed and so the cleaner wrasse does not pass the mark test; the second (II) that cleaner wrasse pass the mark test and are therefore self-aware; and the third (III) that cleaner wrasse pass the mark test, but this does not mean they are self-aware.

If the reader takes position (I), rejecting the interpretation that these behaviours are self-directed, it should be necessary to justify the grounds for this rejection. As noted above, touching or scraping behaviour is taken as evidence of a self-directed behaviour in mammals, and so if these and other behaviours are not similarly considered self-directed in fish, the question must be asked why. For a test to be applicable across species, an objective standard is required. What behavioural criteria need to be fulfilled to define self-directedness in a fish? What is the definition of contingency testing in animals with vastly divergent sensory ecologies? How do we determine an animal is visually exploring its own body when its visual system is nothing like our own? Without such a standard, the behaviours shown in the mark test can be differently assessed depending on the taxon being investigated. This introduces an impossible and unscientific standard for comparison that can never be resolved by debate among differing subjective opinions and therefore undermines the value of the mark test as a comparative tool. This may be an inherent difficulty in comparative studies of animal behaviour, but we do not consider it intractable. Rather, we see great value in computational approaches to behavioural analysis [55] (S5 Video), allowing researchers to decompose behaviour into constituent elements and ask, e.g., whether some kinematic signatures of behaviour are only observed during specific periods of the mark test, or to compute the visual field and determine whether an animal is truly able to see its own reflection. This may allow an objective standard for assessing whether behaviours are unusual, idiosyncratic, or contingent based on quantitative rather than qualitative analysis. It would at the very least provide a quantitative basis for categorisation of different behaviours and thereby facilitate comparison and discussion.

Alternatively, if the behaviours reported here in cleaner wrasse are accepted as being functionally equivalent to those in other taxa during the mark test, position (II) or (III) must be taken. The original interpretation of the mark test by its inventor Gallup posits that species passing the mark test are self-aware [1,56]. A strict adherence to this interpretation would logically lead us to take position (II), that cleaner wrasse are also self-aware. This would require a seismic readjustment of our cognitive *scala naturae*. We are more reserved with our interpretation of these behaviours during the mark test with respect to self-awareness in animals and therefore take position (III). We do not consider, even if our observations are taken as successful behavioural responses to all phases of the mark test, that this should be taken as evidence of self-awareness in the cleaner wrasse. Rather, we consider the interpretation that makes fewest assumptions to be that these fish undergo a process of self-referencing [32,57], in which direct or indirect (e.g., in a mirror reflection) observations of the physical self are perceived as part of one’s own body by the observer but without this involving theory of mind or self-awareness [32,57].

Our conclusion is therefore that cleaner wrasse show behavioural responses that fulfil the criteria of the mark test as laid out for other animals, but that this result does not mean they are self-aware. This position raises a number of difficult questions. Can passing the mark test be taken as evidence of self-awareness in one taxon but not another? We argue not, because a position that holds the same results in a standardised test can be interpreted different ways depending on the taxon from which they are gathered is both logically untenable and taxonomically chauvinistic [58]. Are we instead mistaken in our conclusion that these behaviours even fulfil the criteria of the test? If so, this ambiguity suggests the mark test needs urgent re-evaluation in the context of comparative cognition studies. Finally, while we make no claims that our study proves fish are self-aware, we do hope our results ignite further discussion of fish as cognisant, intelligent animals.

*If a fish is judged by its ability to climb a tree, it will live its whole life believing it is stupid*.—paraphrased from a quote misattributed to Albert Einstein.

## Materials and methods

### Ethics statement

All experiments were conducted in compliance with the animal welfare guidelines of the Japan Ethological Society and were specifically approved by the Animal Care and Use Committee of Osaka City University.

### Animals and housing

The cleaner wrasse, *L*. *dimidiatus*, is a protogynous hermaphrodite teleost that lives in coral reef habitats [46,59]. We used 10 wild fish obtained from commercial collectors in this study. Prior to our experiments, the fish were housed in separate tanks (45 cm × 30 cm × 28 cm), and each fish was kept for at least 1 mo prior to beginning the experiments to ensure acclimation to captivity and the testing conditions and that they were eating and behaving normally. Fish were between 51–68 mm in length; this is smaller than the minimum male size, thus strongly suggesting that these individuals were functionally female. Individual fish sizes were as follows: 68 mm for fish #1, 62 mm for fish #13 and #20, 61 mm for fish #21, 58 mm for fish #4, 55 mm for fish #5, 53 mm for fish #6, 52 mm for fish #2 and #7, and 51 mm for fish #3). Each tank contained a 5 cm × 5 cm × 10 cm rock in the corner and a PVC pipe that provided shelter on a coral-sand substrate 3–4 cm deep. The water was maintained at 24°C–26°C and was aerated and filtered. The fish were maintained on a 12 h:12 h light/dark cycle. Artificial flake food (Tetramin USA) and small pieces of diced fresh shrimp were given twice daily.

### Mirror presentation to fish

The mirror presentation method (e.g., duration, timing, position, and mirror size and shape) has important consequences for successful implementation of MSR studies [1,4,5]. We presented a 45 cm × 30 cm high-quality mirror on a glass wall of the same size inside the experimental tank. The mirror was positioned at night, while the fish were sheltered within the PVC pipe, 1 wk before beginning the experiments; it was then completely covered with a white plastic sheet (45 cm × 30 cm). At the start of the experiments, the white cover on the mirror was removed, and the subject fish were exposed to the mirror until the end of the series of experiments, with the exception of a half-day experiment during which the mirror was completely covered with the white sheet (see below).

### Order of presentation of the five experiments, E1–E5

We studied fish behavioural responses during five experimental periods, from experiment 1 (E1) to experiment 5 (E5; in chronological order). All behaviours were recorded by video camera (HDR-CX370; Sony, Tokyo, Japan), and the field of view encompassed the entire experimental tank. After the start of the experiment (i.e., uncovering of the mirror), the behavioural responses of fish were video recorded eight times over a 2-wk period: every day for the first 5 days and then again on days 7, 10, and 15 (period E1: initial responses to mirror). Three or four days after the end of period E1, fish behaviours were recorded for two consecutive days as a control for the mark test (E2: no mark). Two or three days after E2, a nonvisible sham mark (white or nonpigmented elastomer) was made on all 10 fish, and behavioural responses were recorded over the next two days (period E3: sham marking). Two days after period E3, the fish were marked with a coloured (brown) elastomer mark at night, and their behaviours were recorded in the morning of the following day while the mirror was covered with the 45 cm × 30 cm white plastic sheet (period E4: colour mark with no mirror present). After this observation phase, the mirror was uncovered, and behaviours were recorded on the afternoon of the same day and also on the morning of the following day (period E5; colour mark with mirror present). All experiments were necessarily run in this order because coloured tags could not be removed from fish once applied; hence, transparent (sham) marks always preceded coloured marks. The four fish that were marked on the head showed an increase in scraping of the marked area during period E5. However, three of these fish were also observed scraping facial areas prior to colour marking, indicating that face scraping cannot be taken as unequivocal evidence of mirror-induced self-directed behaviour.

### Provisioning mark procedure

Elastomer tags and visible implant elastomer (VIE) marking (Northwest Marine Technology Inc., Shaw Island, USA) via subcutaneous injection are widely used in studies of individually marked live fish and do not affect fish behaviour [51–54, NMTI]. Our fish were taken from their tanks at night together with their PVC pipe and placed in eugenol solution to achieve mild anaesthesia (using FA100; Tanabe Pharmacy, Tokyo, Japan). A nonpigmented gel mark was injected subcutaneously in an area of 1 mm × 2 mm at one of three sites during the sham mark period: on the right side of the head (two fish), on the left side of the head (two fish), or under the throat (four fish). The entire injection process took no longer than 5 min, and the fish were returned to their original tank together with the pipe after the mirror was covered with the white plastic sheet. We ensured that the fish were swimming normally the next early morning and that they showed no behavioural changes as a consequence of the tagging procedure. We initially used white pigment on the pale-coloured body areas but found that the skin in these areas had a slight blue tint and that the white tag was visible in two fish; these fish were not used in further experiments. A brown-pigmented elastomer colour mark was applied as a colour mark at night before the day of E4. After confirming that all marks were of the same size (1 mm × 2 mm), the fish were returned to the tank. Given the location of the tags relative to the field of view of cleaner wrasse, direct observation of the marks on the head was unlikely and was definitely impossible for throat marks. To standardise the testing procedure, the brown-coloured mark was injected at the throat directly adjacent to the transparent marked site. Even with both marks applied, the total volume of the tag was lower than the minimum recommended amount, even for small fish, and <13% of the size of tags used in studies with other fish (biologists who applied VIE to small fish in previous studies, i.e., 26-mm brown trout [51] and 8-mm damselfish [54], stated that the amounts used were minute, but for the former species, 2–3 mm tags were made with 29 G needles [51]. Willis and Babcock used large tags (10 mm × 1 mm × 1 mm [127/ml]) in *Pagrus auratus* [53]). Our own tagging method was therefore very unlikely to have caused irritation. Moreover, we saw no evidence during period E4 (colour tag, no mirror present) of any removal attempts or scratching behaviour, further confirming that the tags did not stimulate the fish to perform any of the behaviours we report.

### Behavioural analyses

Videos were observed for all behavioural analyses. Fish performed mouth-to-mouth fighting frequently during period E1, and the duration of this behaviour was recorded (Fig 1 and S1 Video). Unusual behaviours performed in front of the mirror, which have never been observed before in a mirror presentation task nor in the presence of a conspecific, were often observed during the first week of E1, and the type and frequency of these behaviours was recorded.

### Description of postural behaviours performed in front of the mirror and behavioural observations

In the latter half of E1, fish occasionally swam slowly or remained stationary in front of the mirror, and the duration (in seconds) of these behaviours, when performed within 5 cm of the mirror, was recorded. The duration of postures in which the marked area was reflected in the mirror was recorded during E2 (no mark), E3 (sham mark), and E5 (coloured mark with mirror present). Posturing within 5 cm of the mirror was categorised into three types: right-sided posture (i.e., reflecting the right side of the head), left-sided posture (reflecting the left side of the head), and frontal–vertical posture (reflecting the throat). The duration (in seconds) of each of the three types of posture was recorded during six separate 5-min observation periods for a total of 30 min per fish for each of the periods when a mirror was present (E2, E3, and E5). A subset of 15% of the videos was blindly analysed by two researchers outside our team; their analysis was highly correlated with the main analysis (*r* = 0.887, *P* < 0.0001), and statistical tests showed no significant differences between the two data sets (two-way repeated-measures analysis of variance [ANOVA], blind effect: *F* = 0.06, *P* = 0.80; blind effect × observation site: *F* = 0.77, *P* = 0.45). Scraping behaviour, including the location on the body that was scraped, was recorded during periods E2–E5 when it occurred. During period E5, when the fish were colour marked and exposed to the mirror, individuals often displayed the marked site to the mirror immediately prior to and following a scraping behaviour. Therefore, we also recorded the time interval between displaying and scraping during E5 (S1 Text).

### Statistical analyses

Statistical analyses were performed using SPSS (ver. 12.0; SPSS Inc., Chicago, IL, USA) and R software (ver. 2.13.2; R Development Core Team 2011). During period E1, the responses of the subject fish to the exposed mirror changed significantly over time. Changes in the duration of mouth fighting and time spent within 5 cm of the mirror over time were analysed with linear mixed models (LMMs). Similarly, changes over time in the duration of mouth fighting and time spent within 5 cm of the mirror were analysed with LMMs for the experiments using real fish across glass dividers. The frequency of unusual mirror-testing behaviours was analysed using a generalised linear mixed model (GLMM) with a log–link function and assuming a Poisson distribution. Time spent in postures reflecting the right side of the head, the left side of the head, and the throat were compared between mark types during the mark tests (E2: no mark, E3: sham mark, and E5: coloured mark with mirror present) using repeated-measures ANOVA. Note that the marked and unmarked positions were analysed separately. Individual-level statistics on postures that reflected the marked sites are shown in Table 2 (Mann–Whitney *U* test with duration in seconds of the six different behaviours per 5-min observation in periods E2, E3, and E5). To detect the effect of throat marking on the frequency of scraping behaviour, a Friedman test was used on the entire data set (E5 versus E2, E3, and E4) and a binomial test was used for comparison between periods (E5 versus E2, E3, and E4). No throat scraping or unusual behaviours were observed when individuals interacted with conspecifics across a glass divider, so no statistical tests were performed for that condition.

## Supporting information

S1 Text Supplementary methods and results. Details of additional methods and results of control experiments.
(DOCX)

S1 Fig The frequency of scraping by a focal animal marked directly on the body. Frequency of scraping before marking (control), after transparent marking (sham), and colour marking (mark) over 3 h in the absence of a mirror. Sham and colour marks were on left flank, an area directly visible for fish (*χ*^2^ = 12.35, df = 2, *n* = 5, *P* < 0.002).
(TIFF)

S1 Data Supporting data. Excel spreadsheet containing behavioural observation data, including initial responses to the mirror, posturing, scraping, and control body mark data.
(XLSX)

S1 Video Mouth fighting against the mirror reflection. The fish attacks the reflection with an open mouth in a common display of fish aggression.
(MOV)

S2 Video Upside-down swimming. The fish approaches the mirror while swimming upside-down.
(MOV)

S3 Video Scraping of the face region. This behaviour was performed against the coral gravel substrate but was also performed against a rock.
(MOV)

S4 Video Scraping of the throat. This behaviour was performed against the coral gravel substrate but was also performed against a rock.
(MOV)

S5 Video Machine-learning tracking of wrasse interacting with a mirror. Preliminary example video of computational approaches to objectively quantify behaviour.
(MOV)

## Abbreviations

ANOVA: analysis of variance
df: degrees of freedom
GLMM: generalised linear mixed model
LMM: linear mixed model
MSR: mirror self-recognition
VIE: visible implant elastomer
