# Supplementary material for: If a fish can pass the mark test, what are the implications for consciousness and self-awareness testing in animals?
Source: PLoS Biol. 2019 Feb 7;17(2):e3000021. doi: 10.1371/journal.pbio.3000021 (PMC6366756; doi:10.1371/journal.pbio.3000021)
Supplement: S1 Text — Details of additional methods and results of control experiments. (DOCX) [file pbio.3000021.s001.docx]

**S1 Text. Supplementary methods and results**. Details of additional methods and results of control experiments.

**Temporal link between scraping and posturing**

In all cases (*n* = 37), scraping behaviours followed soon after the fish had assumed a posture that reflected the throat mark, with an average latency between observation in the mirror and scraping of the substrate of 1.93 sec ± 1.16 (*n* = 37). However, posturing was not always followed by scraping. The physical substrate on which fish scraped varied among individuals: all scraping was done in a narrow area of the sandy bottom by fish #1, and all and the majority (14/16) of the scraping of fishes #21 and #4 was done on a rock in the corner of the tank despite the same arrangement in all tanks. This may be because fish learn successful scraping techniques associated with specific substrates and continue to use them.

**Fish that failed the test**

Fish #20 in our study was throat-marked but did not perform throat scraping (Fig 3A). However, this fish exhibited intensive unusual behaviours (*a*) and (*b*) during period E1, prior to colour-marking, similar to the other fish (Table 1), and assumed postures that reflected the throat more frequently during E5 after colour marking (Table 2), and these results may be interpreted as this wrasse having recognised the reflection as self, but “fell at the last hurdle”. Nevertheless, given the controversial nature of the mark test in non-primates, and questions over the interpretation of the behaviours we observe [e.g. 8], we make no claims on the performance of this individual. We do point out, however, that if we applied the same criteria used for e.g. dolphins, we would necessarily conclude that all four throat-marked fish recognised their reflections in the mirror.

**Responses towards real fish**

A potential alternative explanation of behaviour in mark tests (and one that is rarely tested for in other vertebrates) is that the focal individual perceives their reflection not as self, but rather as another individual behind a glass divide. Although many behaviours seen in the mark test suggest that this is not the case (e.g. contingency-testing, body exploration), and a growing body of evidence shows that fish perceive mirror reflections in a fundamentally different way to conspecifics behind glass [61,62], we directly controlled for this possibility by comparing the behaviour of fish confronted with a reflection to that when another individual was across a glass divide.

We tested the responses of eight fish (55–59 mm in size) in size-matched pairs. Two fish were introduced into a tank (45 × 30 × 26 cm^3^) and after the fish became acclimated, the cover was removed from the divider to allow them to see one another. We then recorded behavioural responses in the same manner as described for period E1 in the mark test, for two weeks. The results of these observation are presented in Fig 1. After three weeks, we marked one of the individuals on the throat and recorded whether the unmarked fish, that could see the marked conspecific through the glass divide, performed any of the unusual behaviours or scraping attempts we observed in the mirror test. We did not observe any instances of these behaviours for any fish. This supports the view that the fish were attempting to remove the mark from their own bodies when presented with the mirror during period E5 of the actual mark test, rather than communicating with a conspecific that it had a parasite (which would involve a similarly high or even higher socio-cognitive process) or the sight of a mark on another individual triggering an innate response to a perceived parasite in the unmarked individual.

**Responses to a directly visible mark on the body and a mark on the mirror itself**

To test for direct self-referencing behaviour, we examined whether wrasse responded to marks on their own flank in an area that was able to be seen by the fish without the aid of a mirror. Five individuals of the same size range as the original experiment were kept in aquaria without mirrors, and their behaviour was recorded for three hours in each of three conditions in the order i) no marking [control], ii) a transparent sham-mark on the centre of the left flank, directly visible for the fish, and iii) a coloured mark on the left flank. The results are shown in S1 Fig. Both sides of the body were infrequently scraped on substrate in the control i) and sham mark ii). In contrast, in the marked phase iii), the marked site on the left flank was frequently scraped, significantly more so than when compared to the right side in the same phase, and the left side of control and sham-marking phases (Interaction: *χ*^2^ = 12.35, *df* = 2, *n* = 5, *P* < 0.002), strongly indicating that when cleaner wrasse can directly see the mark on their own body, they attempt to remove it by scraping behaviour.

This result suggests that cleaner wrasse perceive the coloured marks on the body and attempt to remove them (potentially due to their visual similarity to ectoparasites). We also examined whether cleaner wrasse regard the same marks elsewhere, e.g. on the mirror surface, as ectoparasites and attempt to remove them. We video-recorded and examined their responses of four other fish to a colour mark placed on mirror itself on the first day and ten days after initial mirror presentation (because in the full mark tests, fish performed removal attempts on day ten after passing through the pre-phases). None of the fish approached or directly interacted with the coloured marks on the mirror, nor did they attempt to bite or scrape the marked area of the mirror.

**Visual and tactile stimuli by the colour mark**

We further considered whether the elastomer tag could provide a tactile stimulus that, when paired with visual information, may lead to individuals passing the mark test [8,55]. We can effectively rule out that tactile stimulation alone was sufficient to induce a self-directed behaviour because we observed no throat scraping in any fish during periods E3 (sham mark) and E4 (coloured mark with no mirror present). This is in contrast to previous studies on rhesus monkeys using a somatosensory training stimulation in which the stimulus immediately elicited a response due to irritation [10,11] – we observed no such spontaneous response to the tactile stimulus. Moreover, and likely due to the inherent tendency of the species to search for ectoparasites on the body surface of clients and attempt to remove them [37,45], we did not observe a temporal progression in behaviour that may suggest direct or indirect association learning [8,10,56], rather a rapid onset of removal attempts in period E5. We therefore conclude that the behaviours we observe were primarily driven by visual stimulation, and required no association learning, but acknowledge the provision of elastomer tags may provide more tactile stimulation than paint marks [e.g. 1,4]. Future studies should attempt to experimentally disentangle these two stimuli to assess the roles of visual and tactile stimulation in mark tests with the cleaner wrasse.

**Fish that failed pre-phases**

Although we conclude that three fish immediately failed the pre-phases of the mark test, an alternative explanation is that these fish had already passed through the initial phases before the intended start of the experiment. In these tanks the glass wall on the opposite side of the mirror was slightly reflective due to differences in lighting in the room, and the focal fish were observed to occasionally remain in front of the glass wall. These observations suggest that these three fish may have already passed through phases (*i*) and (*ii*) during the acclimation phase before the start of experiment. These three fish were responsive to the mark test.

**References**

55. Soler M, Pérez-Contreras T, Peralta-Sánchez JM. Mirror-mark tests performed on Jackdaws reveal potential methodological problems in the use of stickers in avian mark-test studies. PLOS One. 2014; 9: e86193.

56. Epstein R, Lanza RR, Skinner BF. “Self-Awareness” in the pigeon. Science. 1981; 212: 695-696.

61. Balzarini V, Taborsky M, Wanner S, Koch F, Frommen JG. Mirror, mirror on the wall: the predictive value of mirror tests for measuring aggression in fish. Behav Ecol Sociobiol. 2014; 68: 871-878.

62. Desjardins JK, Fernald RD. What do fish make of mirror images? Biol Lett. 2010; 6: 744-747.
