# Supplementary figures and images for: If a fish can pass the mark test, what are the implications for consciousness and self-awareness testing in animals?
Source: PLoS Biol. 2019 Feb 7;17(2):e3000021. doi: 10.1371/journal.pbio.3000021 (PMC6366756; doi:10.1371/journal.pbio.3000021)

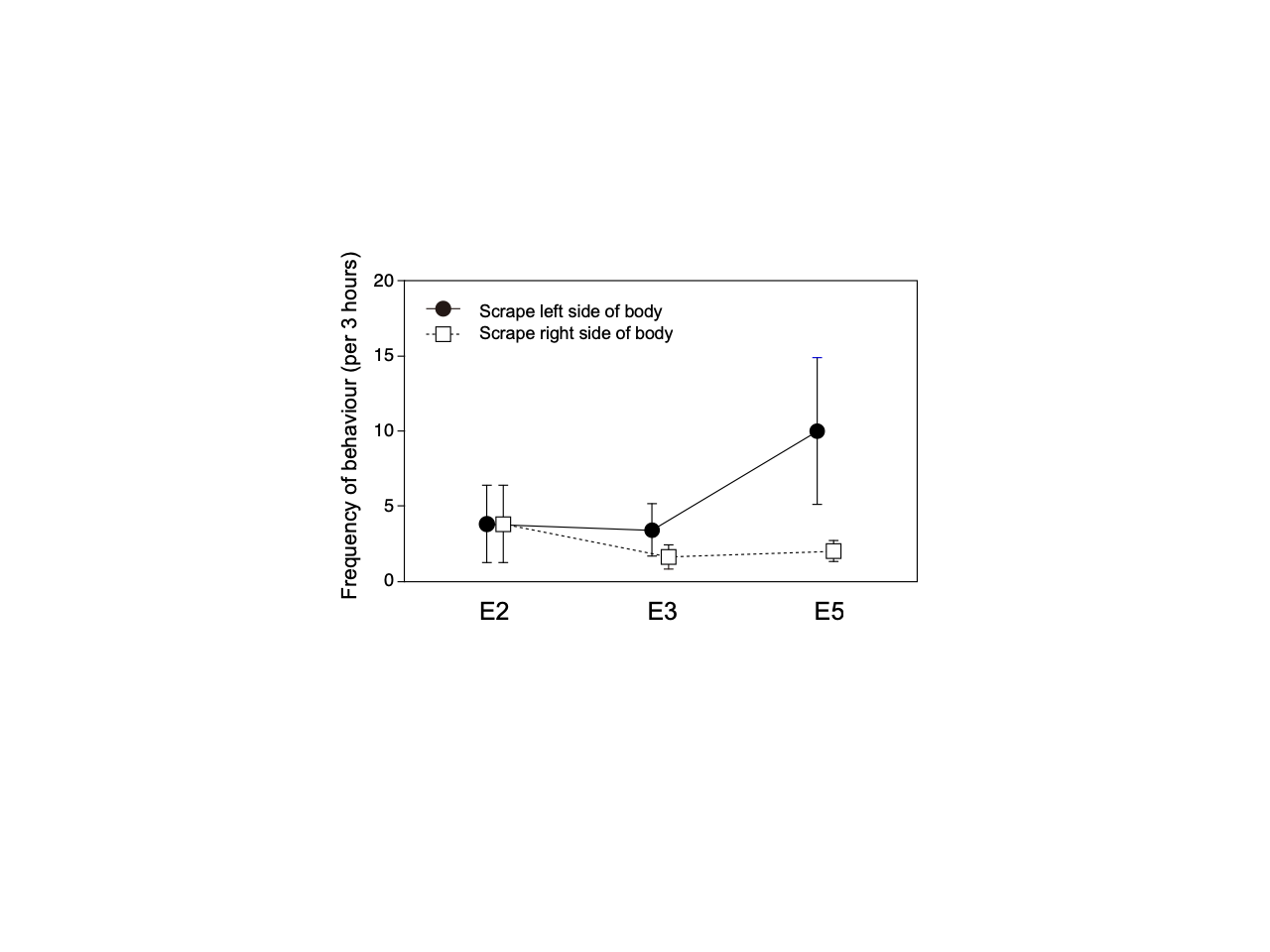

Supplement: S1 Fig — Frequency of scraping before marking (control), after transparent marking (sham), and colour marking (mark) over 3 h in the absence of a mirror. Sham and colour marks were on left flank, an area directly visible for fish (χ2 = 12.35, df = 2, n = 5, P < 0.002). (TIFF) [file pbio.3000021.s002.tiff]
